# Minimally invasive distal radius plating: are outcomes better?

**DOI:** 10.1186/1753-6561-9-S3-A37

**Published:** 2015-05-19

**Authors:** Tatsuji Fujiwara

**Affiliations:** 1 Department of Orthopaedic Surgery, Osaka General Medical Center, Osaka, 534-0021, Japan

## Introduction

Recently, the technique of minimally invasive plate osteosynthesis (MIPO) can also be applied to volar locking plates in distal radius fractures. The advantages of MIPO are fracture healing, a quick return to activities of daily life, and cosmetics as a result of the preservation of the soft tissue compared with conventional plate osteosynthesis. The purpose of the present study was to test the hypothesis that using MIPO for distal radius fractures would achieve better radiologic, clinical, and cosmetic outcomes in volar locking plate fixation compared with conventional methods in consecutive patients performed by a single surgeon in one institution.

## Methods

A total of 31 consecutive patients treated by a single surgeon at one institution for distal radius fractures were included in this study. 31 cases were submitted for pair comparison with consistent gender, age, follow up periods and AO fracture classification. Objective and subjective functional results (range of motion, grip strength, operative time, DASH score, Mayo wrist score, cosmetic satisfaction) and radiographic assessment (volar tilt, radial inclination, ulnar variance, gap and step-off of articular surface in cases of intra-articular fractures, healing time) were assessed. It has been described that it is important for minimizing flexor tendon damage to cover the distal edge of the plate by repositioning intermediate fibrous tissue and pronator quadratus. However, the repositions were not performed because they were impossible due to small incision in our MIPO technique. Thus we assess the relationship between the FPL tendon and the volar locking plate by ultrasonography. Results: An earlier recovery of range of motion was demonstrated in the case of volar flexion and supination up to 1 month after surgery in MIPO group. Operation times for MIPO are longer than that for conventional technique. There was no significant difference between the two surgical techniques in other outcomes. Ultrasonography assessment demonstrated shorter distance between the FPL tendon and the volar locking plate in MIPO than that in the conventional method.

## Conclusions

The MIPO technique is not distinctively superior to conventional technique in main outcomes. On the contrary, the risk of FPL tendon rupture was strongly indicated in MIPO technique from the result of ultrasonography.

